# Decreased synapse‐associated proteins are associated with the onset of epileptic memory impairment in endothelial *CDK5*‐deficient mice

**DOI:** 10.1002/mco2.128

**Published:** 2022-06-20

**Authors:** Zheng‐Mao Li, Xiu‐Xiu Liu, Chen Li, Zhao‐Cong Wei, Yi Shi, Heng‐Yi Song, Xiang Chen, Yu Zhang, Jia‐Wei Li, Rui‐Fang Zhu, Ben‐Hui Hu, Wei‐Feng Ye, Da Huo, Guo‐Jun Jiang, Takuya Sasaki, Li Zhang, Feng Han, Ying‐Mei Lu

**Affiliations:** ^1^ Key Laboratory of Cardiovascular & Cerebrovascular Medicine School of Pharmacy Nanjing Medical University Nanjing China; ^2^ Department of Physiology Nanjing Medical University Nanjing China; ^3^ The First Clinical Medical College of Nanjing Medical University Nanjing Medical University Nanjing China; ^4^ Key Laboratory of Clinical and Medical Engineering School of Biomedical Engineering and Informatics Nanjing Medical University Nanjing China; ^5^ Department of Pharmacy The Children's Hospital, Zhejiang University School of Medicine, National Clinical Research Center for Child Health Hangzhou China; ^6^ Department of Pharmacy Zhejiang Xiaoshan Hospital Hangzhou China; ^7^ Department of Pharmacology Graduate School of Pharmaceutical Sciences Tohoku University Sendai Japan; ^8^ Institute of Brain Science The Affiliated Brain Hospital of Nanjing Medical University Nanjing China; ^9^ Gusu School Nanjing Medical University, Suzhou Municipal Hospital, The Affiliated Suzhou Hospital of Nanjing Medical University Suzhou China

**Keywords:** endothelial CDK5, mechanism, memory impairment, spontaneous epilepsy, synapse‐related proteins

## Abstract

Accumulating evidence indicates that epilepsy has a higher risk of inducing memory impairment and dementia. However, the underlying onset mechanism remains unclear. Here, we found that mice with spontaneous epilepsy induced by endothelial CDK5 deficiency exhibited hippocampal‐dependent memory impairment at 6 months of age, but not at 2 months of age. Moreover, the persistent epileptic seizures induce aberrant changes in phosphorylation of CaMKII protein in the hippocampus of spontaneous epileptic mice. Using genome‐wide RNA sequencing and intergenic interaction analysis of STRING, we found that in addition to epilepsy‐related genes, there are changes in synaptic organization pathway node genes, such as *Bdnf* and *Grin1*. The synapse‐related proteins by Western blot analysis, such as NMDA receptors (NR1 and NR2B), PSD95, and the phosphorylation of synapsin1, are progressively decreased during epileptic seizures in *Cdh5‐CreERT2;CDK5^f/f^* mice. Notably, we found that valproate (VPA) and phenytoin (PHT) augment mRNA expression and protein levels of synapse‐related genes and ameliorate memory impairment in *Cdh5‐CreERT2;CDK5^f/f^* mice. Our study elucidates a potential mechanism of memory deficits in epilepsy, and pharmacological reversal of synaptic pathology targeting might provide a new therapeutic intervention for epileptic memory deficits.

## Graphical abstract

The mechanism of cognitive deficits in epilepsy remains unclear. Here, we found that persistent epileptic seizures induce cognitive deficits by cognitional behavior analysis. Accordingly, changes of synapse‐related gene (*Phka1*, *Btaf1*, *Magt1*, and *Grin1*) and aberrant alternation of NMDA receptors, PSD95, phosphorylation of CaMKII were found in the hippocampus of spontaneous epilepsy mice. More importantly, antiepileptic drugs treatment increased synaptic protein expression and partly ameliorated cognitive dysfunction.

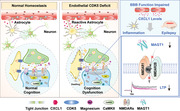

## 1 INTRODUCTION

Epilepsy is one of the most common and disabling neurological disorders.^1^, ^2^ Decreased memory has long been recognized as an epileptic comorbidity, which can occur in children with epilepsy and is more common in clinically refractory epilepsy.^3^, ^4^ Elderly individuals with new‐onset epilepsy are also at a higher risk of developing dementia, especially those with cerebrovascular diseases.^5^, ^6^ It has been reported that cognitive impairment in epilepsy is usually related to potential etiology, onset age, location and frequency, as well as antiepileptic drugs. Nevertheless, less is known about the underlying mechanism of cognitive impairment during epilepsy.

As is widely acknowledged, the hippocampus stands as an essential region responsible for the formation and consolidation of memory, which is an important cognitive process.^7^, ^8^, ^9^ The decreasing activation of mossy cells, which are glutamatergic cell populations in the hilum of the hippocampal dentate gyrus, is sufficient to hamper spatial contextual coding and induce memory deficits in chronically epileptic mice.^10^ Normal synaptic transmission plays a key role in long/short‐term memory formation,^11^, ^12^ and synaptic alterations are reported in epileptic patients and mouse model.^13^, ^14^ However, whether these changes are a cause or a consequence of epilepsy remains a mystery. Moreover, levetiracetam, as an antiepileptic drug, was reported to have a beneficial effect on cognitive networks in drug‐resistant temporal lobe epileptic patients by impeding abnormal network activation.^15^ However, the mechanism by which epilepsy contributes to hippocampal memory dysfunction remains unclear.

Recently, our previous studies have shown that brain endothelial *CDK5* deletion induces progressive reactive astrogliosis and results in the development of spontaneous epilepsy in *Cdh5‐CreERT2;CDK5^f/f^* mice.^16^ Spontaneous epileptic mice show decreased astrocytic GLT1‐mediated currents through endothelial chemokine (C‐X‐C motif), ligand 1 (CXCL1), and its receptor chemokine receptor 2 (CXCR2) in astrocytes. In the present study, LoxP/Cre strategy was used to generate endothelial‐specific *CDK5* knockout mice, and our data indicated that the mice exhibited spontaneous epilepsy accompanied by memory impairment at 6 months old. Our transcriptomic analysis indicated that synapse‐related genes and synapse‐related proteins (PSD95, NR1, and NR2B subunits of NMDA receptors) were decreased in spontaneously epileptic mice. Remarkably, the decreased expression of the NR1 and NR2B subunits of NMDA receptors was reversed by VPA and PHT co‐administration, which was associated with coordinated alleviation of memory impairment in spontaneous epileptic mice. In summary, potent pharmacological interference and synaptic function improvement may help prevent memory impairment during vasogenic epilepsy, potentially providing an important strategy for the treatment of this devastating disease.

## 2 RESULTS

### 2.1 The risk of cognitive dysfunction or dementia in patients with epilepsy

**FIGURE 1:**
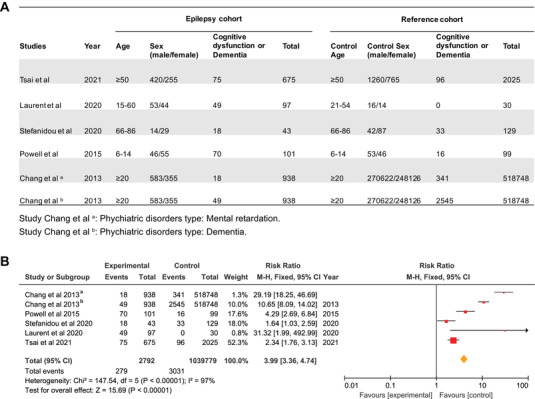
The risk of cognitive dysfunction or dementia in patients with epilepsy. (A) Characteristics of the analytic sample. (B) Results from meta‐analyses of observational and trials about the impact of associations between epilepsy and memory dysfunction or dementia analyzed through RevMan 5 software

To clarify the correlation of epilepsy and memory dysfunction or dementia, a summary of all the meta‐analyses reports from epilepsy is provided in (Figure 1A). For the correlation of epilepsy and memory dysfunction or dementia, the meta‐analysis of the five case–control studies^17^, ^18^, ^19^, ^20^, ^21^ showed that the relative risk (RR) of developing memory dysfunction or dementia was 3.99 times higher (95% CI: 3.36–4.74) in epileptic cohort compared with reference cohort without epilepsy (Figure 1B). The above analysis shows that there is a considerable intersection between epilepsy and cognitive dysfunction, adding that a better understanding of the common mechanisms in these disorders may contribute to the improvement of epileptogenesis and dementia.

### 2.2 Endothelial *CDK5* conditional deletion induces spontaneous epilepsy

**FIGURE 2:**
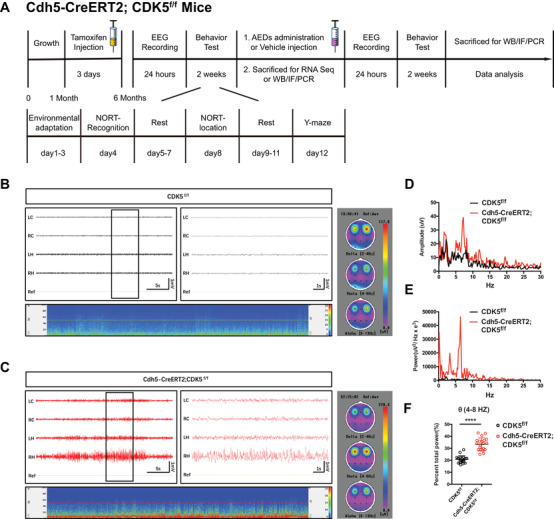
Endothelial *CDK5* deficits caused seizure in mice. (A) The schematic diagram of experimental process. (B and C) Representative EEG recording spectrogram of *CDK5^f/f^* (B) and *Cdh5‐CreERT2;CDK5^f/f^* (C) (LC: left cortex, RC: right cortex, LH: left hippocampus, RH: right hippocampus), oscillation subtypes were distinguished and analyzed, respectively, according to frequency: *δ* (0–4 Hz), *θ* (4–8 Hz), and *α* (8–13 Hz). (D and E) Amplitude (μV) and power (μV^2^/Hz × e^3^) curve were analyzed using Labchart 8 software. (F) Quantitative analysis of single subtype: *θ* (4–8 Hz)/total power ratio. ****p < 0.0001, Student's *t*‐test. Both error bars indicate the mean value ± SEM

To eliminate the effect of gene deficit on development, *Cdh5‐CreERT2;CDK5^f/f^* mice were injected intraperitoneally (IP) with tamoxifen at P30 to induce Cre activity in endothelial cells (ECs) to generate an endothelial *CDK5*‐deficient strain (Figure 2A). EEG recordings for local field potential suggested that epileptic waves were observed in the hippocampal CA1 region of *Cdh5‐CreERT2;CDK5^f/f^* mice at 6 months old but not in the cortex of both CKO and control mice (Figure 2B,C). The amplitude, power density, and percentage of total power (%) were significantly higher at the theta band (4–8 Hz) in *Cdh5‐CreERT2;CDK5^f/f^* mice than in *CDK5^f/f^* mice (Figure 2D–F). Consistent with previous results, these data suggested that endothelial *CDK5*‐deficit strain induced a spontaneous epileptic phenotype in *Cdh5‐CreERT2;CDK5^f/f^* mice.

### 2.3 Progression of epilepsy leads to impaired hippocampal‐dependent memory

**FIGURE 3:**
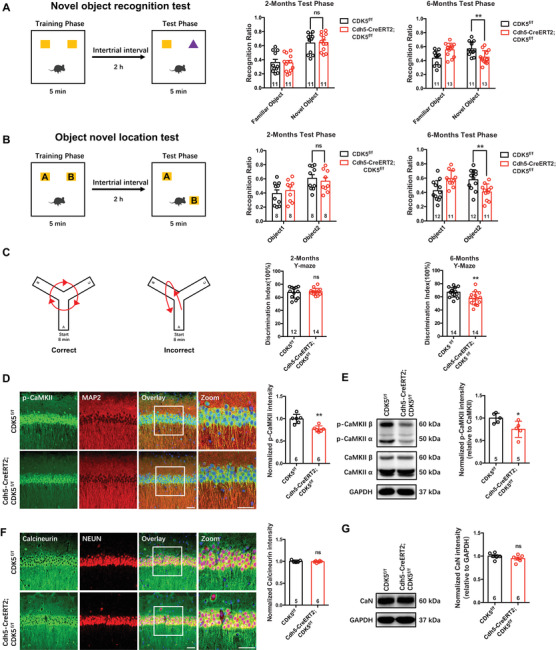
Persistent seizures lead to hippocampus‐dependent memory impairment. (A–C) Hippocampus‐dependent memory behaviors were performed in *CDK5^f/f^* and *Cdh5‐CreERT2;CDK5^f/f^* mice. The schematic diagram of novel object recognition test and the discrimination index value (A), the schematic diagram of object new location memory and the discrimination index value (B), the schematic diagram of Y‐maze and the discrimination index value (C). (D and E) Representative phospho‐CaMKII (p‐CaMKII, Thr286) (green) staining in CA1 of mice (D, left, counterstained with microtubule‐associated protein 2 [MAP2] [red] and nuclear marker DAPI [blue]) and quantification of normalized p‐CaMKII intensity (D, right, **p < 0.01, Student's *t*‐test). Representative immunoblotting bands of p‐CaMKII (Thr286), CaMKII, and GAPDH (E, left), and quantitative analyses by densitometry for p‐CaMKII, *p < 0.05 (E, right). (F and G) Representative calcineurin (CaN) (green) staining in CA1 of mice (F, left, counterstained with neuronal marker [NEUN] [red] and nuclear marker DAPI [blue]) and quantification of normalized CaN intensity (F, right, ns: not significant, Student's *t*‐test). Representative immunoblotting bands of CaN and GAPDH (G, left), and quantitative analyses by densitometry for CaN (G, right). Scale bar: 20 μm, magnified images: 50 μm

To verify the potential concomitant effects of endothelial spontaneous epilepsy, several behavioral tests on memory were performed. We previously reported that *Cdh5‐CreERT2;CDK5^f/f^* mice showed an age‐dependent increase in the prevalence and frequency of seizures. Here, we checked memory behavior at 4 weeks (2‐month‐old mice) and 20 weeks (6‐month‐old mice) after tamoxifen treatment. First, in novel object recognition analysis, the decrease in the discrimination index suggested that the ability of mice to recognize either novel objects or novel locations was also impaired at 6 months old but not at 2 months old in *Cdh5‐CreERT2;CDK5^f/f^* mice (Figure 3A,B). Second, the *CDK5*‐deficient mice also exhibited a significant decrease in discrimination index of Y‐maze test at 6 months old, not at 2 months old (Figure 3C). Finally, the levels of phosphorylated CaMKII (Thr286), a critical signaling molecule in hippocampal memory formation, were also reduced in *Cdh5‐CreERT2;CDK5^f/f^* mice (Figure 3D,E). Calcineurin (CaN), a Ca^2+^‐calmodulin‐dependent serine/threonine protein phosphatase, was not changed in *Cdh5‐CreERT2;CDK5^f/f^* mice (Figure 3F,G). Collectively, these data suggest that endothelial *CDK5* knockout impairs hippocampal‐dependent memory.

### 2.4 Endothelial *CDK5* deficiency causes aberrant changes in synapse‐related genes in the hippocampus

**FIGURE 4:**
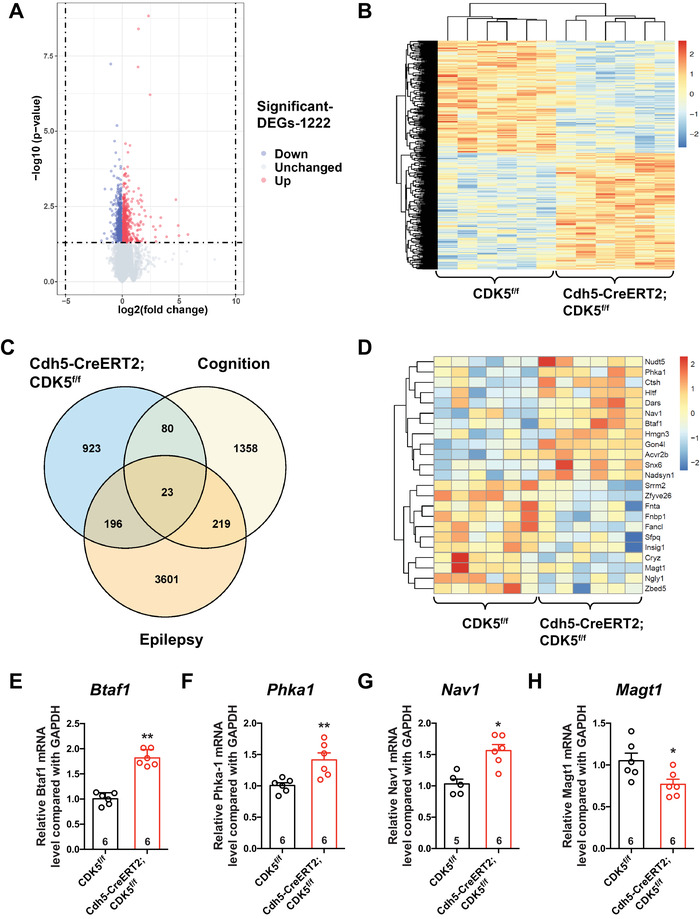
Changes of gene expression in the hippocampus of endothelial *CDK5‐*deficit mice. (A) Volcano plot of genes expression profiler. (B) Heatmap of 1222 differentially expressed genes. (C) Venn plot representing the number of intersecting genes. (D) Heatmap of 23 intersecting genes. (E–H) The relative mRNA level of indicated genes (*Btaf1, Phka1, Nav1, Magt1*) in hippocampus of *CDK5^f/f^* and *Cdh5‐CreERT2;CDK5^f/f^* mice

To inspect the molecular mechanism that contributes to memory impairment, we performed digital gene expression by whole‐genome RNA sequencing and genomics analysis on hippocampal tissue samples obtained from *Cdh5‐CreERT2;CDK5^f/f^* and *CDK5^f/f^* mice. Using gene expression microarrays combined with network analysis, we found 55,573 robustly expressed genes in the transcripts. A total of 1222 differentially expressed genes (DEGs; 577 upregulated genes and 645 downregulated genes) in mutant mice compared to control mice were screened (Figure 4A,B).

To explore the correlation between epilepsy and memory dysfunction caused by *CDK5* deletion in ECs, transcripts were cross‐analyzed with epilepsy (GSE60772) and memory dysfunction (GSE80312) transcripts. Eventually, we sorted 23 target genes (12 upregulated genes and 11 downregulated genes) from the cross‐analysis data (Figure 4C,D). Next, we used quantitative real‐time PCR (RT‐PCR) to confirm the mRNA levels of 23 DEGs filtered from the three transcripts above. The RT‐PCR results suggested that the mRNA levels of *Btaf1*, *Phka1*, and *Nav1*, which possess a strong correlation with epilepsy, were upregulated significantly (Figure 4E–G). Accordingly, the magnesium ion transporter‐encoding gene *Magt1* was observed to have decreased mRNA levels (Figure 4H). Together, the mRNA levels of *Btaf1*, *Phka1*, *Nav1*, and *Magt*1 were related to epilepsy.

**FIGURE 5:**
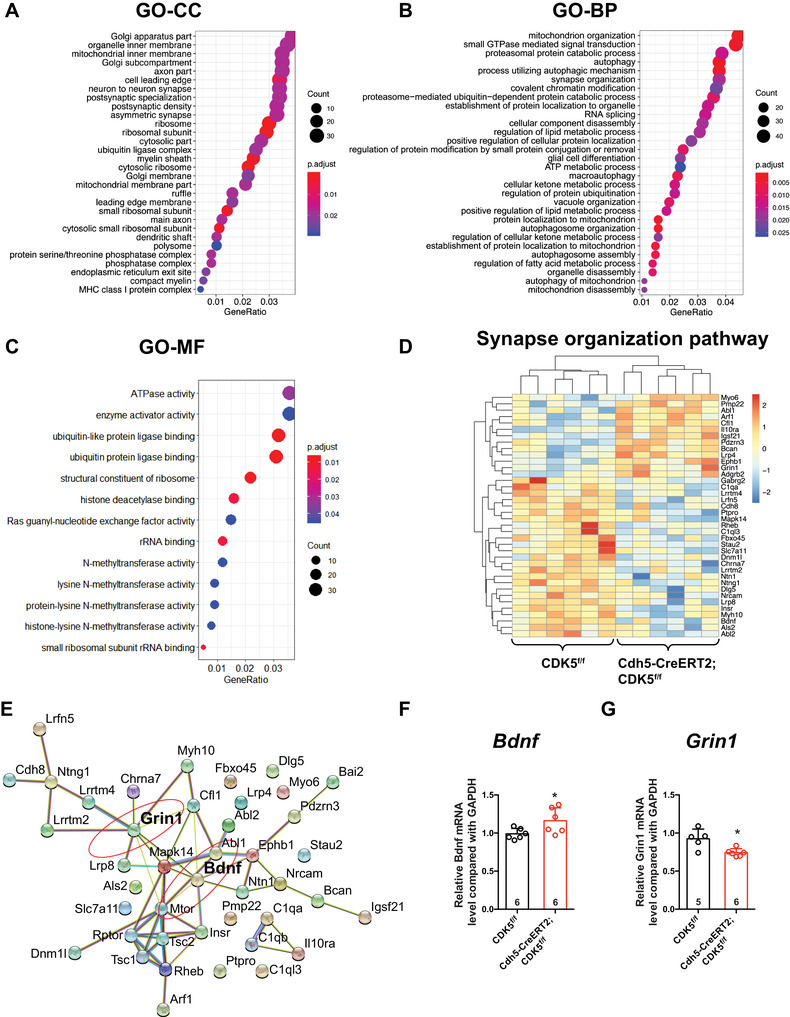
Endothelial *CDK5* deficiency causes changes of synapse‐related gene in hippocampus. (A) Cellular component analysis. (B) Biological process analysis. (C) Molecular function analysis. (D) Heatmap of synapse organization pathway genes. (E) Protein–protein interaction (PPI) network of synapse organization pathway genes. (F and G) The relative mRNA level of *Grin1* and *Bdnf* genes of hippocampus in both strains. *p < 0.05, **p < 0.01, Student's *t*‐test. All error bars indicate the mean value ± SEM

In addition, through the Gene Ontology (GO) database, the differentially expressed genes were classified by cellular component (CC), molecular function (MF), and biological process (BP), and we found that the screened differentially expressed genes were involved in the regulation of neuron‐to‐neuron synapses, postsynaptic specialization, and postsynaptic density (Figure 5A–C). After further enrichment analysis of the synaptic organization pathway by KEGG signaling pathway, 38 differential genes were obtained. Genes involved in the synaptic organization pathway were screened, among which 13 genes were decreased and 25 genes were increased (Figure 5D). Through gene‐to‐gene interaction analysis by STRING, we found that *Bdnf* ^22^, ^23^ and *Grin1* ^24^, ^25^ were two important node genes in the synaptic organization pathway (Figure 5E). *Grin1* mRNA was downregulated in *Cdh5‐CreERT2;CDK5^f/f^* mice, and *Bdnf* mRNA was upregulated (Figure 5F,G). Therefore, out data suggest that aberrant changes in synapse‐related genes in the hippocampus might contribute to the memory impairment in endothelial *CDK5*‐deficiency mice.

### 2.5 Expression of synapse‐related proteins decreased in the hippocampus of *Cdh5‐CreERT2;CDK5^f/f^* mice

**FIGURE 6:**
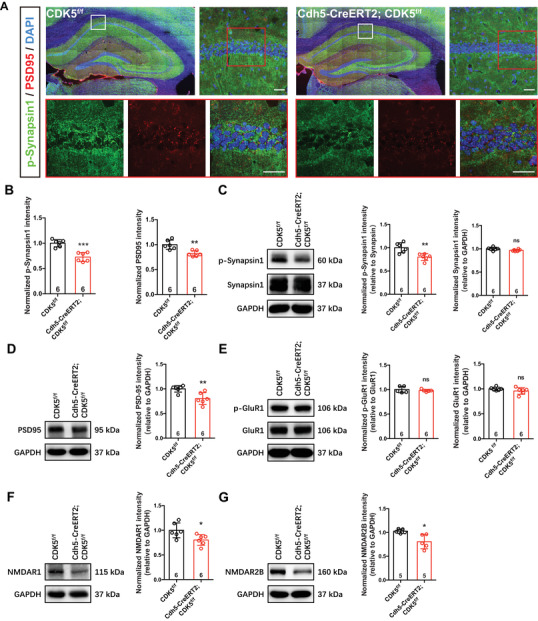
Synapse‐related proteins decreased in hippocampus of *Cdh5‐CreERT2;CDK5^f/f^* mice. (A and B) Representative p‐Synapsin1 (green) and PSD95 (red) staining in hippocampus of mice (A, counterstained with DAPI [blue], top right, scale bar: 20 μm; bottom, scale bar: 50 μm) and quantification of normalized p‐Synapsin1 and PSD95 intensity (B) (**p < 0.01, ***p < 0.001, Student's *t*‐test). (C and D) Representative immunoblots of synapse structure‐related proteins p‐Synapisn1/Synapsin1 (C) and PSD95 (D), and quantitative analyses by densitometry (**p < 0.01, ns: not significant, Student's *t*‐test). (E–G) Representative immunoblots of synapse receptors NMDARs (NR1 and NR2B) (F and G) and GluR1/phospho‐GluR1 (p‐GluR1) (E), and quantitative analyses by densitometry (*p < 0.05, **p < 0.01, ns: not significant, Student's *t*‐test, NR2B, *n* = 5; NR1, GluR1/p‐GluR1). All error bars indicate the mean value ± SEM

To examine whether structural proteins of neuronal synapses were changed in the hippocampus of *Cdh5‐CreERT2;CDK5^f/f^* mice, we analyzed the protein levels of Synapsin1 (a presynaptic marker) and PSD‐95 (a postsynaptic marker in excitatory neurons). The immunofluorescence data showed that the phosphorylation of Synapsin1 and the protein level of PSD95 were significantly decreased (Figure 6A,B). Similar to the immunofluorescence data, the Western blot data showed that the protein level of Synapsin1 had no change, but p‐Synapsin1 significantly decreased in *Cdh5‐CreERT2;CDK5^f/f^* mice at 6 months of age (Figure 6D), which suggested that phosphorylation‐dependent regulation of synaptic vesicle clusters was decreased. Moreover, PSD95 and synaptic receptors (NR1, NR2B) were dampened in *Cdh5‐CreERT2;CDK5^f/f^* mice (Figure 6D,F,G), and GluR1 and p‐GluR1 showed no changes (Figure 6E). However, immunofluorescence staining data show that the number of hippocampal neurons was not changed in the CA1, DG, or CA3 regions (Figure S1A,B). Synaptic activation is a key link in a chain of molecular and biochemical events involved in memory formation.^11^, ^12^ Together, the progression of epilepsy‐impeded setup of synaptic proteins is close correlated with memory impairment in endothelial *CDK5*‐deficiency mice.

### 2.6 Antiepilepsy drugs alleviated seizures, improved memory, and aligned with normalized synaptic protein levels

**FIGURE 7:**
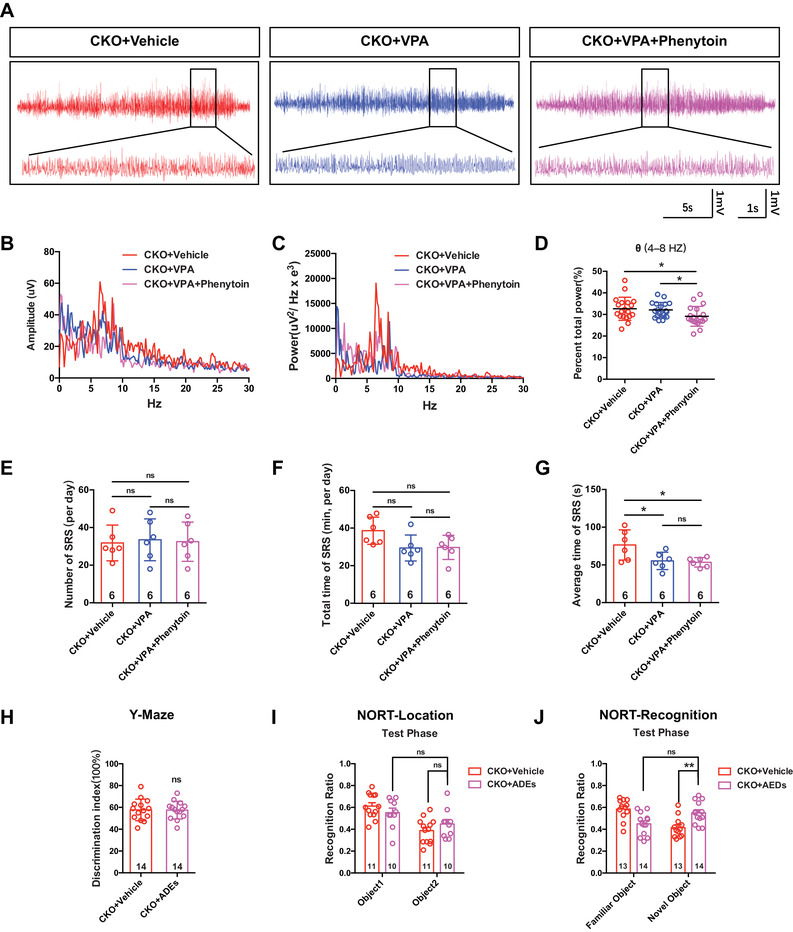
Antiepilepsy drugs alleviated seizure and ameliorate memory. (A) Representative EEG recording spectrogram for 24 h in *Cdh5‐CreERT2;CDK5^f/f^* mice administered with valproic acid (VPA, 300 mg/kg/day) or with VPA (300 mg/kg/day) combined phenytoin (PHT, 30 mg/kg/day) for 7 days consecutively. (B and C) Amplitude (B) and power curve (C) were analyzed using Labchart 8 software. (D) Quantitative analysis of single subtype: *θ* (4–8 Hz)/total power ratio (*p < 0.05, two‐way ANOVA). (E–G) The spontaneous recurrent seizure (SRS) was analyzed using Labchart 8 from aspects of average duration (E), total times per day (F), and total duration per day (G) (E–G, *p < 0.05, ns: not significant, two‐way ANOVA). (H–J) Memory behavioral tests were performed. Y‐maze (H), novel object recognition (I), and object new location recognition (J) (*p < 0.05, **p < 0.01, ns: not significant, (H) Student's *t*‐test, (I and J) two‐way ANOVA). All error bars indicate the mean value ± SEM

VPA is one of the most widely used anticonvulsant and mood‐stabilizing agents for treating epilepsy.^26^ PHT is the recommended second‐line intravenous anticonvulsant for the treatment of pediatric convulsive status epilepticus.^27^ To explore whether VPA and PHT alleviate spontaneous epileptic seizures, EEG recordings were performed after intraperitoneal injection of VPA for 7 consecutive days. The data showed that VPA or VPA+PHT treatment attenuated epileptic seizures in *Cdh5‐CreERT2;CDK5^f/f^* mice, and the power of the theta band (4–8 Hz) was also decreased significantly after VPA or VPA+PHT administration (Figure 7A–D). Although the number of spontaneous recurrent seizures (SRSs) at 24 h and the total time of SRS at 24 h did not change (Figure 7E,F), the average time of SRS changed significantly after VPA or VPA+PHT administration compared to CKO+vehicle, and there was no difference between VPA and VPA+PHT treatment (Figure 7G).

**FIGURE 8:**
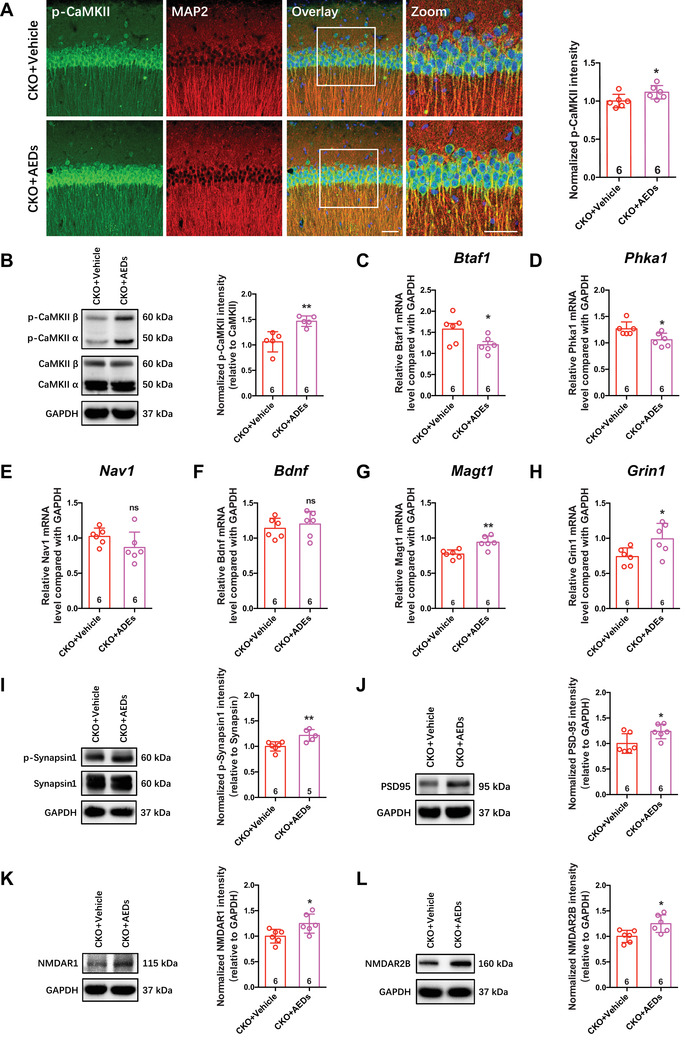
Antiepilepsy drugs increased synaptic proteins level. (A and B) Representative p‐CaMKII (Thr286) (green) staining in CA1 of mice (A, left, counterstained with MAP2 [red] and DAPI [blue], scale bar: 20 μm, magnified images: 50 μm) and quantification of normalized p‐CaMKII intensity (B, right, *p < 0.05, Student's *t*‐test). Representative immunoblotting bands of p‐CaMKII (Thr286), CaMKII and GAPDH, and quantitative analyses by densitometry for p‐CaMKII (B). (C–G) The relative mRNA level of indicated genes in hippocampus. *Btaf1* (C), *Phka1* (D), *Nav1* (E), *Bdnf* (F), *Magt1* (G), and *Grin1* (H) (*p < 0.05, **p < 0.01, ns: not significant, Student's *t*‐test). (I–L) Representative immunoblotting bands of synapse‐related proteins in hippocampus and quantitative analyses by densitometry (*p < 0.05, **p < 0.01, Student's *t*‐test). All error bars indicate the mean value ± SEM

To verify whether antiepileptic drug administration ameliorates memory deficits, we conducted Y‐maze and novel object recognition tests (NORT) after antiepileptic drugs (AEDs, VPA+PHT) treatment. The data showed that AEDs administration improved the NORT object recognition ability (Figure 7J), but had no effects on the Y‐maze or NORT location behaviors (Figure 7H,I). Moreover, the phosphorylation of CaMKII also increased after AEDs treatments, as shown by Western blot and immunofluorescence staining analyses (Figure 8A,B).

Moreover, to clarify the effects of AEDs on the mRNA levels of epilepsy‐related and synapse‐related proteins in *Cdh5‐CreERT2;CDK5^f/f^* mice, the mRNA levels of *Btaf1, Phka1*, *Nav1*, *Bdnf*, *Magt1*, and *Grin1* were confirmed by qRT‐PCR. Antiepileptic drug administration decreased the expression of *Phka1* and *Btaf1* and elevated the expression of *Grin1* and *Magt1* (Figure 8C–H). Finally, we tested the protein concerning synaptic structure and its receptors. Western blot data indicated that AEDs restored the protein levels of the synaptic structure‐related proteins p‐Synapsin1/Synapsin1 and PSD95 and the synaptic receptors NR1 and NR2B (Figure 8I–L).

Hence, AEDs exerted a therapeutic effect on vasogenic epilepsy derived from endothelial *CDK5* loss, which results in downregulating epilepsy‐related *Phka1* and *Btaf1* and upregulating synaptic function‐related *Grin1* and *Magt1*. We speculate that improvement of memory and improved phosphorylation of CaMKII in the hippocampus may be associated with restoration of synaptic structural components p‐Synapsin1 and PSD95, as well as synaptic receptors NR1 and NR2B function.

## 3 DISCUSSION

To date, the mechanism of memory deficits in epilepsy remains unclear. An imbalance of synaptic excitation and inhibition is considered to be the basis of epilepsy; however, whether the imbalance leads to memory impairment is also unclear. Here, clinical meta‐analysis of epilepsy patients showed that the risk of memory impairment or dementia was higher in epilepsy patients than in controls. We utilized the spontaneous epilepsy model to discover that persistent epileptic seizures induce memory deficits by memory behavior analysis. Accordingly, synapse‐related gene and protein changes were found in the hippocampus of *Cdh5‐CreERT2;CDK5^f/f^* mice. More importantly, AEDs treatment increased synaptic protein levels and partly ameliorated memory dysfunction.

In adult hippocampus, *CDK5* modulates synaptic plasticity, memory formation, and long‐term behavioral changes.^28^, ^29^ Neuronal hyperactivation of *CDK5* has been reported to be involved in the pathological process of many neurodegenerative diseases, including AD.^30^, ^31^ Here, we found that endothelial *CDK5*‐deficit mice exhibited memory impairment and decreased phosphorylation of CaMKII after tamoxifen treatment for 20 weeks and not for 4 weeks. Long‐term potentiation (LTP) has an important role in hippocampus‐dependent learning and memory and plays a basic role in memory processes.^32^, ^33^ CaMKII is a key protein kinase in hippocampal neural plasticity and memory.^34^, ^35^ The enzyme CaMKII can be autophosphorylated at T286 on the alpha subunits and at 287 on the beta subunits,^36^ which makes CaMKII activity persist even after the calcium concentration drops to baseline levels.^37^ The persistent activation of CaMKII is sufficient to trigger LTP, and CaMKII activation is necessary for LTP. In epileptic *Cdh5*‐*CreERT2;CDK5^f/f^* mice, although the total protein level of CaMKII had no change and no neurons were lost, autophosphorylated CaMKII decreased, which may play an important role in memory deficits caused by epilepsy.

NMDARs, as glutamate receptors, are blocked by Mg^2^^+^ ions, which are only removed when neurons depolarize. MagT1 is located in the cell membrane and facilitates Mg^2+^ influx, which maintains intracellular Ca^2+^ levels and protects neurons from hyperexcitatory Ca^2+^ overload.^38^ Here, we showed that the decreased mRNA level of *MagT1* may contribute to seizures in *CDK5*‐deficient mice. On the other hand, LTP maintenance requires different downstream molecular events, such as CaMKII activation, PSD, NR2B, NR1, and AMPAR.^39^ Activated CaMKII diffuses to the synapse and specifically binds to NR2B, which plays a key role in LTP induction and learning.^40^, ^41^ Moreover, PSD95, which is present in excitatory synapses, stabilizes the surface expression of NMDAR and is necessary for CaMKII,^42^ NMDAR, and CaMKII–NMDAR complex binding in the postsynaptic membrane.^43^ In the present study, transcriptomics analysis revealed synaptic gene changes, which were also verified by decreased protein levels of PSD95, NR1, and NR2B in tamoxifen‐treated *Cdh5‐CreERT2;CDK5^f/f^* mice. Decreased protein levels of PSD95, NR1, and NR2B affect the formation and maintenance of hippocampal neural plasticity, resulting in memory impairment. The ATP release/CD73/A2AR pathway preceded and related with synaptic dysfunction and neuronal damage.^44^ Therefore, it is of interest to further address the role of metabolic changes in the context of vasogenic epilepsy in the future.

PHT and VPA are clinical first‐line antiepileptic drugs. PHT is used to control seizures by modulating voltage‐gated sodium channels,^45^ and VPA is effective in most seizure types.^46^ According to our study, VPA treatment, or combination with PHT, could decrease epileptic gene mRNA expression and alleviate seizures. VPA is approved for the treatment of mania associated with manic depression, epileptic seizures, and migraine headaches.^26^ However, the mechanism of VPA remains unknown. Interestingly, we found that VPA treatment increased the phosphorylation of CaMKII and the protein levels of NR2B, NR1, and PSD95, which may contribute to improve memory behaviors. Nevertheless, the mechanism of VPA on synapses needs to be explored in the future.

In summary, our data indicated that the mice exhibited memory impairments in the process of spontaneous epilepsy. To explore the cellular and molecular characteristics underlying spontaneous epileptic memory impairments, we generated a transcriptomic atlas of the hippocampus in *CDK5^f/f^* mice and *Cdh5‐CreERT2;CDK5^f/f^* mice. We identified the transcriptional alterations underlying the dysregulation of epilepsy‐ and synapse‐related gene changes in the hippocampus and unveiled synapse‐related protein aberrations associated with epileptic memory impairments. Moreover, VPA or PHT co‐administration rescued impaired hippocampal synaptic plasticity, resulting in memory improvement. Overall, our study describes a mechanism of memory deficits in epilepsy and identifies new effects of VPA on synapses, which provides new insights into preventive or therapeutic interventions for epileptic memory deficits. In the future, the study of the neuronal circuit mechanism of memory dysfunction in vasogenic epilepsy and the pharmacological regulation of targeting this neural circuit will promote the treatment of this disease.

## 4 MATERIALS AND METHODS

### 4.1 Experimental animals

Mice were housed under the condition of a 12‐h light/dark schedule with a controlled atmospheric environment (22°C ± 1°C, 40%–60% humidity) and had free access to standard diet and water. Certain experimental operations were applied to the animals following at least 1 week of acclimation to their habitat. All animal studies and experimental procedures were approved by the Animal Experimentation Committee at Nanjing Medical University in China (1804007).

The generation of endothelial‐specific *CDK5* knockout mice applied LoxP/Cre strategy. To construct *Cdh5‐CreERT2;CDK5^f/f^* mice, endothelial tamoxifen‐inducible driver *Cdh5‐CreERT2* mice (acquired from Prof. Ralf Adams, Max Planck Institute, G¨ottingen, Germany) were crossed with mice with a loxP‐flanked *CDK5* gene (stock no. 014156; The Jackson Laboratory). Cre activity would be induced at 4 weeks by tamoxifen injections (Sigma‐Aldrich, 0.1 mg/g bodyweight, intraperitoneal), as described previously.^16^

### 4.2 Behavioral analysis

Mice at 4 weeks (2‐month‐old mice) and 20 weeks (6‐month‐old mice) after tamoxifen treatment were used for the experimental procedures. Homologous *CDK5^f/f^* mice were treated as the control group. Y‐maze, novel object location test, and novel object recognition test were performed.

#### 4.2.1 Y‐maze test

Spontaneous alternation behavior of *Cdh5‐CreERT2;CDK5^f/f^* mice relative to *CDK5^f/f^* mice was recorded for spatial memory evaluation in the Y‐maze task, as described previously.^47^ Briefly, mice were placed at the end of one arm of the apparatus with three identical black plexiglass arms (length: 50 cm, width: 16 cm, height: 32 cm) to allow them to freely explore the three arms of the maze during an 8‐min session. An alternation was defined by every three different choices through recording the sequence of arm entries visually. The calculation of discrimination index (%) was (actual alternations/maximum alternations) × 100.

#### 4.2.2 Novel object recognition test

An object recognition task on the basis of the mice tending to discriminate between familiar and novel objects was carried out according to a previously validated method to evaluate recognition memory.^48^ Two same objects (A and B) were placed in symmetric positions in the chamber (50 × 50 × 60 cm) center. During the acquisition phase training, mice were put into the chamber to explore the objects (A and B) for 5 min. Two hours later, exploratory behavior was assessed for 5 min after a novel object C replacing object B. The discrimination index (%) was calculated as Times of exploring novel (C)/Total times of exploring (A+C).

#### 4.2.3 Object novel location test

To test the spatial memory of mice, two same objects (A and B) were placed in symmetric positions in the chamber (50 × 50 × 60 cm) center. During the acquisition phase training, mice were made to explore in the chamber for 5 min. The position of object B was changed to the diagonal area of object A 2 h after the acquisition phase training, then the mice were put into the chamber to explore the objects for 5 min. The discrimination index (%) was calculated as Times of exploring novel (B)/Total times of exploring (A+B).

### 4.3 EEG recording

EEG recording could be used to monitor spontaneous seizure activity as reported previously.^16^ In brief, bipolar twisted stainless steel electrodes (0.2 mm in diameter; Plastics One) were placed bilaterally in hippocampus CA1 region (−2.0 mm rostral to bregma, ±1.5 mm to midline, and 1.5 mm ventral to bregma) of the mice. Then, the electrodes were connected into a hole through a plastic pedestal (6 Channel, Plastics one), followed by being fixed to the skull with dental cement. After a week of recovery, EEG recording was conducted using a Vanguard system (Lamont) continuously in freely moving mice at a sampling rate of one utilizing a high‐frequency filter of 70 Hz for 24 h. The definition of epileptic seizures was field potentials two‐fold greater than the basal potential following durations longer than 10 s. Raw recording data were analyzed by Labchart 8 software.

### 4.4 Drugs administration

VPA (valproic acid sodium salt, Sigma P4543) and PHT (phenytoin sodium, Sigma D4505) were procured from Sigma‐Aldrich, Co., St. Louis, MO, USA. After EEG recording, the epileptic mice were screened for intraperitoneal injection of VPA (300 mg/kg/day) individually or VPA (300 mg/kg/day) + PHT (30 mg/kg/day) combined administration for 7 days consecutively. Then, EEG and memory‐related behavioral tests were performed.

### 4.5 Immunohistochemistry

Immunohistochemistry was performed as previously described.^47^ After fixation in 4% PFA at 4°C, overnight, the brain was fully preserved in 30% sucrose PBS solution for cryoprotection. Then, 40‐μm slices from the brains were cut in a frozen microtome equipment later. The slices were permeabilized with 0.1% Triton X‐100 for 15 min, then blocked in 5% bovine serum albumin (BSA) for another hour at room temperature. For immunolabeling, the slices were incubated with indicated primary antibodies: anti‐CaMKII (1:300, Fukunaga et al., 1988), anti‐phospho‐Thr286‐CaMKII (1:300, Fukunaga et al., 1988), anti‐calcineurin (1:300, Fukunaga et al., 1988), anti‐MAP2 (1:1000, Millipore, Cat# 05‐346), anti‐NEUN (1:500, Millipore, Cat# ABN78), anti‐phospho‐Ser603‐Synapsin1 (1:300, Millipore, AB5583), and anti‐PSD95 (1:300, Thermo, MA1‐045). After incubation for two nights at 4°C, slices were incubated with Alexa Fluor 488‐conjugated anti‐mouse IgG (1:300, A21202, Life Technologies) and Alexa Fluor 594‐conjugated anti‐rabbit IgG (1:300, A21207, Life Technologies) for 1 h at RT. Using Zeiss LSM 800 confocal microscope for images and fluorescence values and co‐localization were later analyzed with ImageJ software.

### 4.6 Western blot analysis

Western blot analysis was performed as previously described.^49^ It has been reported that memory spatiotemporal alterations manifest differently in circuits in different subregions of the hippocampus, but are fully integrated in the CA1 neuronal network as independent, multiplexed representations.^50^ Therefore, we extracted total extracts of the whole tissue of the hippocampal CA1 region for the detection of related protein indicators. In brief, samples containing equivalent amounts of protein were separated by SDS‐PAGE (10%–12%) and transferred onto PVDF membranes (Millipore). The primary antibodies were listed as follows: phospho‐Thr286‐CaMKII (1:3000, Fukunaga et al., 1988), CaMKII (1:3000, Fukunaga et al., 1988), anti‐phospho‐Ser603‐Synapsin1 (1:1000, Millipore, AB5583), anti‐Synapsin1 (1:1000, Fukunaga et al., 1988), anti‐PSD95 (1:5000, Thermo, MA1‐045), anti‐NMDAR1 (1:1000, Abcam, ab109182), anti‐NMDAR2B (1:1000, Alomone, AGC‐003), anti‐phospho‐Ser831‐GluR1 (1:1000, Millipore, 04‐823), anti‐GluR1 (1:1000, Santa Cruz, SC‐7608), and GAPDH (1:5000, Cell Signaling Technology, Cat# 2118). EZ‐ECL Chemiluminescence Detection Kit was used to visualize immunoreactive proteins (Biological Industries). Quantification of the bands was performed using ImageJ software (US National Institutes of Health). GAPDH was used as an internal control.

### 4.7 RNA‐seq data analysis

According to false discovery rate (FDR), differentially expressed genes (DEGs) were selected (value <0.05). Heatmaps were produced using the R package heatmap. FPKM values were *z*‐scaled and used as input data for heatmap. We used R package Venn diagram to fetch the intersection of *CDK5* CKO sequencing data, GSE80312 and GSE128300.^51^, ^52^, ^53^ Gene Ontology‐biological process (GO‐BP) and Gene Ontology‐cellular component (GO‐CC) terms were enriched and visualized using the R package cluster Profiler.^54^ *p*‐Value <0.05 was set as the cutoff value for the enrichment analysis. STRING database (www.string‐db.org) was used to predict protein–protein interaction (PPI) network of DEGS of synapse organization term.^55^

### 4.8 Real‐time quantitative PCR

In order to validate the mRNA levels of differentially expressed genes obtained by RNA‐seq analysis, brain hippocampus tissues were dissected. The total RNA of the samples was extracted using RNAiso Plus (Takara, Shiga, Japan), as described previously.^56^ First strand cDNA was synthesized by reverse transcription with 500 ng total RNA using the Prime Script RT reagent Kit Perfect Real Time (Takara) driven by indicated primer pairs in a 20‐μl reaction system according to the manufacturer's instructions. qRT‐PCR was performed with a Mastercycler ep realplex (Eppendorf, Hamburger, Germany) in 96‐well plates using SYBR Premix Ex Taq (Takara) to detect the mRNA expression level of 23 DEGs. The primer sequences are provided in Table S1. PCR condition is 95°C for 2 min, 40 cycles of each 95°C for 15 s, 55°C for 15 s, and 68°C for 25 s. Melting curve analysis was performed with the default settings on the instrument from 50°C to 85°C. Commercial software was used to calculate the RNA expression *C*_t_ values automatically. β‐actin or GAPDH was used as an internal control. Data were expressed relative to a calibrator using the 2‐(DD*C*_t_) ± s formula.

### 4.9 Statistical analysis

Statistical analysis was performed using GraphPad Prism 6 (GraphPad Software, San Diego, CA, USA). An unpaired two‐tailed Student's *t*‐test was used for datasets, including two mere independent groups. For multigroup comparisons, one‐way ANOVA was performed by Tukey's multiple comparison test. Two‐way ANOVA followed by Bonferroni's multiple comparison test was performed. Data results were presented as means ± SEM and *p* < 0.05 was considered to be statistically significant.

## CONFLICT OF INTEREST

The authors declare no competing interests.

## AUTHOR CONTRIBUTIONS

ZM.L., XX.L. contributed equally to this work, conducted experiments, analyzed data and write articles, C.L., ZC.W., Y.S. and HY.S. conducted behavioral analysis and data collation, X.C., Y.Z., JW.L., RF.Z. analyzed of transcriptomic data, BH.H., WF.Y., D.H., GJ.J and T.S. assisted with the experiments and reviewed paper. L.Z., F.H., YM.L. designed and coordinated the experiments, supervised the work, reviewed and edited paper.

## ETHICS APPROVAL

All animal studies and experimental procedures were approved by the Animal Experimentation Committee at Nanjing Medical University in China (1804007).

## Supporting information

SUPPORTING INFORMATION

## Data Availability

The RNA‐seq data generated in this study are available at NCBI’s Gene Expression Omnibus GEO database (https://www.ncbi.nlm.nih.gov/geo/query/acc.cgi; accession # GSE198170). The other datasets are available from the corresponding authors upon reasonable request.
