# Supplementary material for: Decreased synapse‐associated proteins are associated with the onset of epileptic memory impairment in endothelial CDK5‐deficient mice
Source: MedComm (2020). 2022 Jun 20;3(3):e128. doi: 10.1002/mco2.128 (PMC9209881; doi:10.1002/mco2.128)
Supplement: Supplementary file 1 — SUPPORTING INFORMATION [file MCO2-3-e128-s001.docx]

**Decreased synapse-associated proteins are associated with the onset of epileptic memory impairment in endothelial *CDK5*-deficient mice**

Zheng-Mao Li^1#^, Xiu-Xiu Liu^1#^, Chen Li^1^, Zhao-Cong Wei^2^, Yi Shi^2^, Heng-Yi Song^1^, Xiang Chen^1^, Yu Zhang^1^, Jia-Wei Li^3^, Rui-Fang Zhu^3^, Ben-hui Hu^4^, Wei-Feng Ye^5^, Da Huo^1^, Guo-Jun Jiang^6^, Takuya Sasaki^7^, Li Zhang^8^, Feng Han^1,8,9*^, and Ying-Mei Lu^2,8*^

^1^Key Laboratory of Cardiovascular & Cerebrovascular Medicine, School of Pharmacy, Nanjing Medical University, Nanjing, 211166, China.

^2^Department of Physiology, Nanjing Medical University, Nanjing, 211166, China.

^3^The First Clinical Medical College of Nanjing Medical University, Nanjing Medical University, Nanjing, 211166, China.

^4^Key Laboratory of Clinical and Medical Engineering, School of Biomedical Engineering and Informatics, Nanjing Medical University, Nanjing, 211166, P. R. China.

^5^Department of Pharmacy, The Children’s Hospital, Zhejiang University School of Medicine, National Clinical Research Center for Child Health, Hangzhou, China

^6^Department of Pharmacy, Zhejiang Xiaoshan Hospital, Hangzhou, Zhejiang, China

^7^Department of Pharmacology, Graduate School of Pharmaceutical Sciences, Tohoku University, Sendai 980-8578, Japan.

^8^Institute of Brain Science, The Affiliated Brain Hospital of Nanjing Medical University, Nanjing 211166, China

^9^Gusu School, Nanjing Medical University, Suzhou Municipal Hospital, The Affiliated Suzhou Hospital of Nanjing Medical University, Suzhou, 215002, China.

^#^The authors contributed equally to this manuscript.

^*^ Corresponding authors:

Dr. Feng Han. Tel: 86-25-8686-8462, E-mail: fenghan169@njmu.edu.cn;

Dr. Ying-Mei Lu. Tel: 86-25-8686-9495, E-mail: lufx@njmu.edu.cn

**Supplementary Materials**

**Table 1 The primer sequence of the differential genes obtained by RNA-Seq**

**screening.**


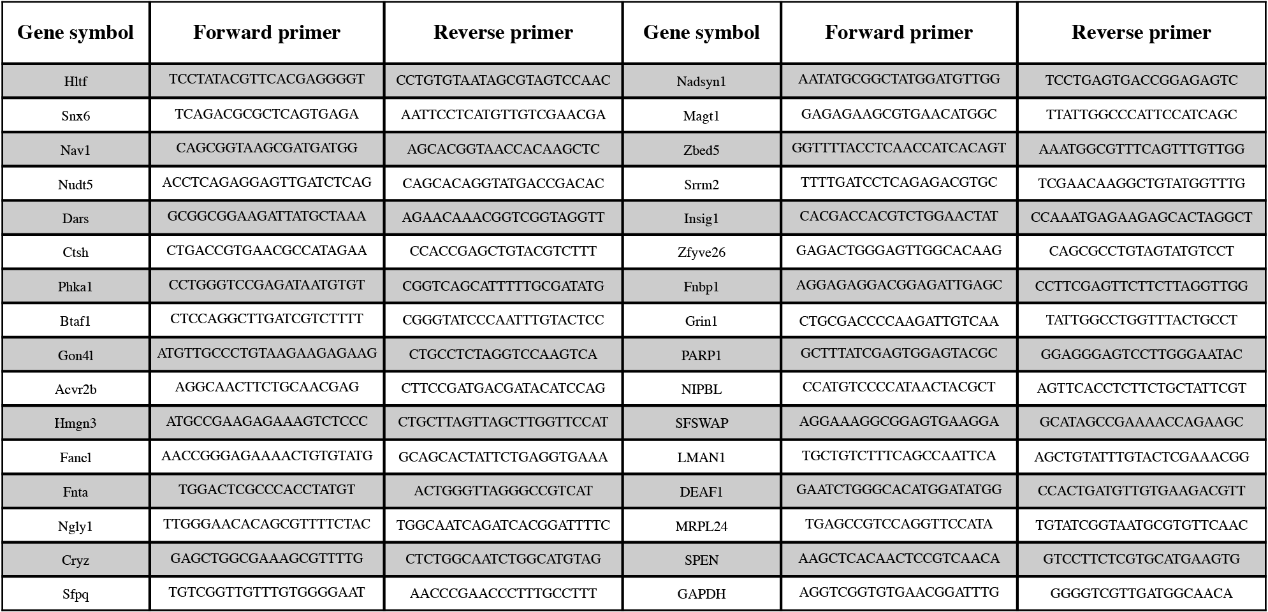


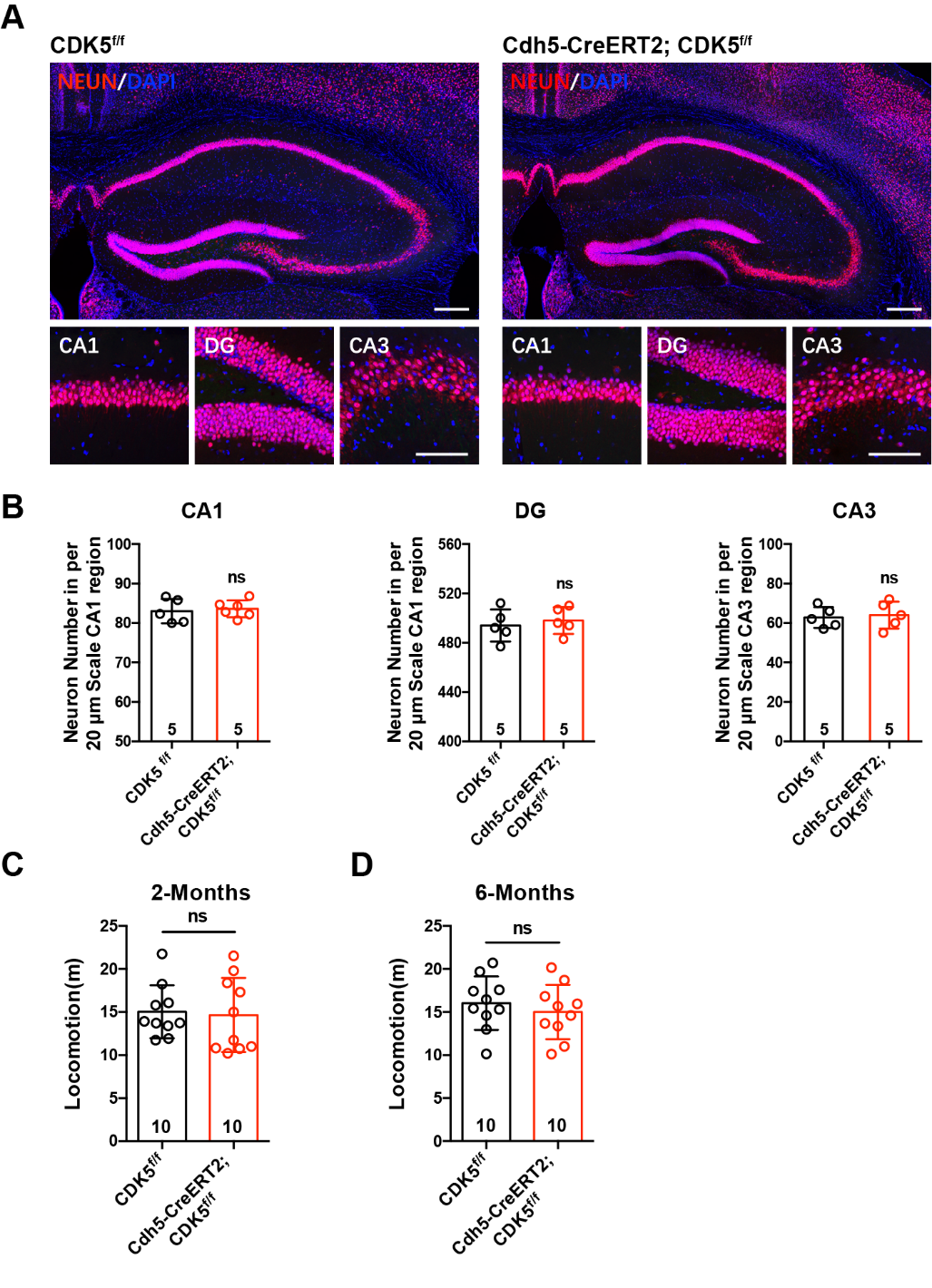


**FIGURE S1 Endothelial CDK5 deficiency did not reduce hippocampal neurons in mice.** Representative NEUN staining (A) and quantification of normalized NEUN intensity (B) in CA1, DG and CA3 regions of mice. (C-D) Total distance statistics in of 2-month-old and 6-month-old mice in free station. Above scale bar, 20μm, below scale bar, 50μm, ns: Not significant.
